# Recent Advances in Maize Nuclear Proteomic Studies Reveal Histone Modifications

**DOI:** 10.3389/fpls.2012.00278

**Published:** 2012-12-12

**Authors:** Paula Casati

**Affiliations:** ^1^Centro de Estudios Fotosintéticos y Bioquímicos, Universidad Nacional de RosarioRosario, Santa Fe, Argentina

**Keywords:** *Zea mays*, nuclei, histones, posttranslational modification, mass spectroscopy

## Abstract

The nucleus of eukaryotic organisms is highly dynamic and complex, containing different types of macromolecules including DNA, RNA, and a wide range of proteins. Novel proteomic applications have led to a better overall determination of nucleus protein content. Although nuclear plant proteomics is only at the initial phase, several studies have been reported and are summarized in this review using different plants species, such as *Arabidopsis thaliana*, rice, cowpea, onion, garden cress, and barrel clover. These include the description of the total nuclear or phospho-proteome (i.e., *Arabidopsis*, cowpea, onion), or the analysis of the differential nuclear proteome under different growth environments (i.e., *Arabidopsis*, rice, cowpea, onion, garden cress, and barrel clover). However, only few reports exist on the analysis of the maize nuclear proteome or its changes under various conditions. This review will present recent data on the study of the nuclear maize proteome, including the analysis of changes in posttranslational modifications in histone proteins.

## Introduction

The eukaryotic nucleus is highly dynamic and complex, it has several subcompartments, types of DNA and RNA, and a wide range of proteins. Different novel proteomic applications have led to a better overall determination of nucleus protein content, enabling researchers to analyze protein–protein interactions, structures, activities, and even posttranslational modifications (Erhardt et al., 2010).

Although nuclear plant proteomics is only at the initial phase, several studies have been reported using different plants species. However, there are a low number of publications in the field using maize (*Zea mays*). These low numbers of publications have also mainly focused on a small number of nuclear proteins, the histones. Because insights from any species can trigger ideas for research in other plants, and due to the lack of research in this area at present on maize nuclear proteomics and its limited scope focused around histones, in this review, examples of experiments completed using different plant species are presented, including recent data on the study of the nuclear maize proteome, and in particular the histones.

## Examples of Plant Nuclear Proteome Analysis

One example of an analysis of changes in the nuclear proteome, and particularly in the phospho-proteome of a plant species was done using onion nuclei (González-Camacho and Medina, 2004). To detect variations associated with cell proliferation, two-dimensional proteomes from soluble fractions of onion nuclei isolated from actively proliferating meristematic and non-meristematic root cells were compared (González-Camacho and Medina, 2004). Interestingly, the nucleolin-like protein NopA100 was significantly increased in proliferating cells, and Western blots with anti-NopA100 antibody demonstrated 26 spots in the meristematic sample corresponding to this protein. All the spots detected were clustered at 100 kDa, suggesting NopA100 was differentially phosphorylated, and that the protein was more highly phosphorylated in cycling cells (González-Camacho and Medina, 2004).

The *Arabidopsis* nuclear proteomes of plants under different growth conditions has also been analyzed. In response to cold stress, 54 out of 184 protein spots were changed in response to cold treatment in two-dimensional gels (2D-PAGE; Bae et al., 2003); while in the presence of oligogalacturonides, elicitors of plant defense responses, significant changes in protein abundance for 19 proteins were reported (Casasoli et al., 2007). Proteins responding to the oligogalacturonide treatment were involved in the protein translation machinery and regulation, suggesting a general reprogramming of the plant cell metabolism in response to oligogalacturonides (Casasoli et al., 2007). The proteome of the *Arabidopsis* nucleoli was also analyzed. The eukaryotic nucleolus is involved in ribosome biogenesis and a range of other RNA metabolism and cellular functions; in this compartment 217 proteins were identified by proteome analysis (Pendle et al., 2005). The comparison of the proteomes of the arabidopsis and the human nucleoli identified many common proteins, plant-specific proteins, proteins of unknown function in both proteomes, and proteins that were nucleolar in plants but non-nucleolar in human, suggesting that in plants, nucleoli may have additional functions in mRNA export or surveillance (Pendle et al., 2005).

A proteome reference map of a legume, chickpea, was completed using 2D-PAGE (Pandey et al., 2006). Approximately, 600 protein spots were detected and LC-ESI-MS/MS analyses led to the identification of 150 proteins that have been implicated in different cellular functions. These included proteins involved in signaling, gene regulation, DNA replication, and transcription (Pandey et al., 2006). Besides, the nuclear proteome of chickpea seedlings under dehydration conditions was compared to that of control plants using 2D-PAGE (Pandey et al., 2007). MS analysis allowed the identification of 147 differentially expressed proteins involved in various functions, including gene transcription and replication, molecular chaperones, cell signaling, and chromatin remodeling (Pandey et al., 2007). A similar study was done using a draught tolerant rice variety, identifying 150 proteins that showed changes in their levels (Choudhary et al., 2009). The proteomic analysis led to the identification of differentially regulated proteins involved in transcriptional regulation and chromatin remodeling, signaling and gene regulation, cell defense and rescue, and protein degradation. Furthermore, a comparison between the dehydration responsive nuclear proteome of rice and that of chickpea, showed an evolutionary divergence in dehydration response, with only a few conserved proteins (Choudhary et al., 2009). Using rice, a nuclear proteome analysis was used to search for novel nuclear proteins that could play evolutionarily conserved roles in the sugar response in plants (Aki and Yanagisawa, 2009). Five hundred sixty-three different proteins were identified by nanoLC/ESI/MS/MS analysis of extracts from rice nuclei that were purified by Percoll density gradient centrifugation, whereas 307 different proteins were identified with nucleic acid-associated proteins that were enriched by DNA affinity chromatography (Aki and Yanagisawa, 2009). Among them, transcription and splicing factors were identified, but also a mediator of sugar signaling in plants, hexokinase.

The nuclear proteome of *Medicago truncatula* 12 days after pollination (dap) was also analyzed; this stage marks the switch toward seed filling (Repetto et al., 2008). Nano-liquid chromatography–tandem mass spectrometry analysis of nuclear protein bands excised from 1D SDS-PAGE identified 179 polypeptides, providing an insight into the complexity and distinctive feature of the seed nuclear proteome, and highlighting new plant nuclear proteins with possible roles in the biogenesis of ribosomal subunits or nucleocytoplasmic trafficking (Repetto et al., 2008). To identify proteins that contribute to disease resistance in soybean, the nuclear proteome from a susceptible cultivar was compared to that of a resistant inbred isoline (Cooper et al., 2011). About 4975 proteins from nuclear preparations of leaves were detected using a high-throughput liquid chromatography-mass spectrometry method. Statistics of summed spectral counts revealed proteins with differential accumulation changes between susceptible and resistant plants; however, these protein accumulation changes were compared to previously reported gene expression changes and very little overlap was found. Thus, it appears that numerous proteins are posttranslationally affected in the nucleus after infection (Cooper et al., 2011). Finally, the nuclear proteome of the unicellular green alga *Chlamydomonas reinhardtii* was also analyzed (Winck et al., 2012). Using LC-MS/MS, 672 proteins from nuclei isolates were identified. Well-known proteins like histones, transcription factors and other transcriptional regulators were identified (Winck et al., 2012).

Only few reports exist on the analysis of the maize nuclear proteome or its changes under various conditions. Next, we will present recent data on the study of the nuclear maize proteome, including the analysis of changes in posttranslational modifications in histone proteins.

## Maize Nuclear Proteome Studies

A comparison of the maize nuclear proteomes after a UV-B light treatment was done using maize lines that differ in UV-B tolerance by 2D-PAGE (Casati et al., 2008). Eight maize lines with documented differences in UV-B tolerance were employed. The W23 UV-B light-sensitive line, deficient in flavonoid sunscreens, and high-altitude Confite Puneño and Mishca lines selected in their natural environment for UV-B tolerance were compared. In addition, four hypersensitive lines expressing RNAi constructs to reduce the expression of predicted chromatin remodeling genes (*chc101*, *nfc102*, *sdg102*, and *mbd101*) were compared to the B73 line. Fluorescently labeled proteins were resolved by isoelectric focusing on a 3–10 pH gradient and in the second dimension by molecular weight using PAGE. Approximately 500 proteins were resolved; most protein spots were present in all genotypes; there were only a few cases in which spots were present in some lines and not in others (Casati et al., 2008). Differential accumulation of chromatin proteins, particularly histones, constituted the largest class identified by mass spectrometry; other DNA- and chromatin-associated proteins, and several ribosomal proteins were also identified.

UV-B-tolerant landraces and the B73 inbred line showed twice as many protein changes as the UV-B-sensitive W23 line and transgenic maize expressing RNAi constructs directed against chromatin factors (Casati et al., 2008). Although many changes were line-specific, reflecting the distinctive germplasm of the two high-altitude lines and of W23, it was clear that UV-B-tolerant lines exhibit more nuclear proteome changes than do sensitive lines. For example, the high-altitude Confite and Mischa showed 42 and 31 protein changes, respectively, while the sensitive W23 line showed 21 protein spots changed by the treatment. Paralleling the conclusion based on all proteins, more changes in histone proteins were found in the high-altitude lines than in W23. Similarly, more histone isotypes were differentially accumulated in B73 than in the near-isogenic RNAi transgenic lines.

## Analysis of Histones and Histone Covalent Modifications in Maize Nuclei

**Figure 1:**
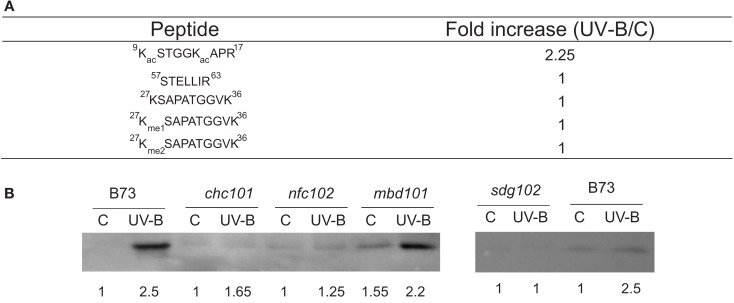
**Analysis of histone H3 modifications in maize after a UV-B treatment**. **(A)** Average ratios of peak areas integrated from LC-MS runs of representative peptides detected from the duplicates of UV-B-treated and the control in the absence of UV-B histone H3. The peptide 9-KacSTGGKacAPR-17 is more abundant in the UV-B-exposed sample, while non-covalent modified or methylated peptides are not changed by the treatment. **(B)** Western blot analysis of histone extracts from maize plants revealed using antibodies against acetylated H3 in the N-terminal domain. Quantification of the bands determined by densitometrical analysis of the western blots is shown below each band. Data adapted from Casati et al. (2008) and Campi et al. (2012).

For some histones, the same protein was identified in different spots in one gel (Casati et al., 2008). The presence of the same protein type at multiple spots could reflect either differential expression of loci encoding different proteins or posttranslational regulation of the same gene product. Histones are subject to numerous covalent modifications, such as acetylation, methylation, phosphorylation, and ubiquitination, and these modifications control many aspects of chromatin function mediated by histones (Kouzarides, 2007). To analyze histone composition systematically by MS, histones were acid extracted from UV-B-treated or control B73 leaves and then the four core histones were separated by reverse phase HPLC. A direct comparison of histones H2B, H2A, H4, and H3 at the protein level did not reveal any noticeable difference between the UV-B-treated and control samples. Nevertheless, substantial changes were observed in some acetylated peptides at the N-terminal tails of H4 and H3. The acetylated H4 N-terminal tail, for example, was approximately doubled in UV-B-exposed samples compared with the control (Casati et al., 2008). Tryptic peptides of histone H3 were analyzed in a similar manner; an N-terminal tail acetylated peptide was observed to be considerably increased in UV-B-treated samples as well (Figure 1A). In contrast, the level of detected methylations remained essentially unchanged after UV-B treatment (Figure 1A). Thus, for both H3 and H4, the differential intensities of various isoforms observed by 2D-PAGE were explained at least in part by posttranslational modification levels that change in response to UV-B treatment, and acetylation in the N-terminal tail of H3 and H4 is the most significantly altered epigenetic mark after UV-B treatment. These acetylated histones were enriched in the promoter and transcribed regions of two UV-B-upregulated genes examined; radiation-sensitive lines lack this enrichment (Casati et al., 2008).

The increase in histone acetylation by UV-B was also demonstrated by Western blot analysis (Figure 1B; Campi et al., 2012). Using antibodies against acetylated histone H3 in the N-terminal domain, maize plants from the B73 genotype showed increased acetylation of this histone after a UV-B treatment, similarly as shown by MS analysis (Figure 1A; Casati et al., 2008). However, the RNAi *chc101*, *nfc102*, *sdg102*, and *mbd101* chromatin remodeling deficient plants showed lower levels of H3 acetylation after the UV-B treatment than wild-type plants (Figure 1B). Together, these experiments demonstrated that chromatin remodeling, and in particular histone acetylation, are important for UV-B responses in maize. In particular, UV-B radiation can also induce different chromatin remodeling events in the promoter regions of *Mutator* transposons (Qüesta et al., 2010). Increased transcript abundance of the *mudrA* transposase and *mudrB*, an unknown gene encoded in *MuDR*, the master *Mutator* transposon, is accompanied by an increase in histone H3 acetylation and by decreased H3K9me2 methylation (Qüesta et al., 2010). To date, only radiation treatments such as UV-B have reactivated silenced *Mutator*. Therefore, transposon reactivation by UV-B requires epigenetic changes, suggesting that early changes in H3 methylation and chromatin remodeling contribute directly to transposon reactivation by UV-B in maize (Qüesta et al., 2010).

So far, changes in histone covalent modifications have been the most extensively studied posttranslational modifications in maize nuclei. A method for the reliable and sensitive detection of specific chromatin modifications on selected genes has been described (Jaskiewicz et al., 2011). The technique is based on the crosslinking of modified histones and DNA with formaldehyde, extraction and sonication of chromatin, chromatin immunoprecipitation with modification-specific antibodies, de-crosslinking of histone-DNA complexes, and gene-specific real-time quantitative PCR. This approach has proven useful for detecting specific histone modifications associated with C4 photosynthesis in maize (Jaskiewicz et al., 2011). In addition, the relative abundance of eight different histone modifications was tested at various regions in several imprinted maize genes using a chromatin immunoprecipitation protocol coupled with quantitative allele-specific single nucleotide polymorphism assays (Haun and Springer, 2008). Imprinting is an epigenetically controlled form of gene regulation in which the expression of a gene is based on its parent of origin. This epigenetic regulation is likely to involve allele-specific DNA or histone modifications. In this work, histone H3 lysine-27 di- and tri-methylation were paternally enriched at three imprinted loci. In contrast, acetylation of histones H3 and H4 and H3K4 dimethylation were enriched at the maternal alleles of these genes. Di- and tri-methylation of H3 lysine-9, which is generally associated with constitutively silenced chromatin, was not enriched at either allele of imprinted loci. These patterns of enrichment were specific to tissues that exhibit imprinting; in addition, the enrichment of these modifications was dependent upon the parental origin of an allele (Haun and Springer, 2008). Changes in the covalent modification of histones were also demonstrated during paramutation (Haring et al., 2010). Paramutation is the transfer of epigenetic information between alleles that leads to a heritable change in expression of one of these alleles. Paramutation at the tissue-specifically expressed maize *b1* locus involves the low-expressing *B*′ and high-expressing *B-I* allele. A hepta-repeat located 100 kb upstream of the *b1* coding region was required for paramutation, and nucleosome occupancy, H3 acetylation, and H3K9 and H3K27 methylation were mainly involved in tissue-specific regulation of the hepta-repeat (Haring et al., 2010).

A different example of a proteomic analysis of histones was done using maize kernels (Kalamajka et al., 2010). In maize kernel development, the onset of grain-filling represents a major developmental switch that correlates with a massive reprogramming of gene expression. In this study, the linker histones of developing maize kernel tissue were compared. Chromosomal linker histones from developing maize kernels before (11 dap) and after (16 dap) initiation of storage synthesis were isolated, and six linker histone gene products were identified by MALDI-TOF mass spectrometry. The acid soluble histones were separated by 2D-PAGE, several of the spots corresponding to the linker histone bands were excised from the gels, in gel digested with trypsin, and examined by MALDI-TOF MS. Peptide fragmentation data were obtained by MS/MS analysis of intense peaks in the MS spectra (Kalamajka et al., 2010). The linker histones HON101, HON102, HON103, HON104, HON106, and HON110 were identified; interestingly, the majority of the linker histones from the 11 dap endosperm were found to migrate in the 2D gels with a lower p*I* than those from the 16 dap endosperm sample. Since the same gene products were identified in gel spots from 11 to 16 dap, the difference in linker histone p*I*s is most likely to be due to differential posttranslational modification(s) of the proteins during kernel development (Kalamajka et al., 2010).

As previously shown, histone modifications have also been analyzed by immunological techniques. To investigate the mitosis-dependent cross-talk between histone H4 tetra-acetylation, DNA methylation, and H3K9 dimethylation, specific antibody immunostaining in Western blot analysis and in *in situ* chromatin immunostaining were used to detect and compare H4ac, H3K9me2, and DNA methylation patterns during mitosis in maize root meristems (Yang et al., 2010). Treatment with trichostatin A, which inhibits histone deacetylases, resulted in increased histone H4 acetylation accompanied by the decondensation of interphase chromatin and a decrease in both global H3K9 dimethylation and DNA methylation during mitosis. These observations suggest that histone acetylation may affect DNA and histone methylation during mitosis. Treatment with 5-azacytidine, a cytosine analog that reduces DNA methylation, caused chromatin decondensation and mediated an increase in H4 acetylation, in addition to reduced DNA methylation and H3K9 dimethylation during interphase and mitosis, suggesting that decreased DNA methylation causes a reduction in H3K9 dimethylation and an increase in H4 acetylation (Yang et al., 2010). Using a DNA fiber-fluorescence *in situ* hybridization approach to study individual maize centromeres, the association of a specific centromeric histone H3 (CENH3) was visualized in centromeres (Jin et al., 2004). This analysis revealed that CENH3 is always associated with centromere-specific satellite repeats, but that not all these sequences are associated with CENH3 (Jin et al., 2004). Finally, fluorescence *in situ* hybridization analysis of a reciprocal translocation in maize between chromosomes 1 and 5 revealed the presence of an inactive centromere at or near the breakpoints of the two chromosomes (Gao et al., 2011). To confirm the active and inactive states of two sets of centromeric sequences, biochemical features of active centromeres were examined in root tip metaphase spreads of this material. CENP-C is an inner kinetochore protein that is characteristic of all active centromeres (Dawe et al., 1999). CENP-C was only located at the site of the primary constriction and was not detectable at the second cluster of centromere sequences. A second biochemical feature of active centromeres is the phosphorylation of Serine-10 on histone H3 (Houben et al., 2007). Immunolocalizations combined with FISH using antibodies against this histone modification revealed that only the set of centromeric sequences at the primary constriction were detectably labeled. Together, the results showed that this centromere does not exhibit any of the tested biochemical features of active centromeres, and it extends the evidence for an epigenetic component to centromere function in plants (Gao et al., 2011).

## Perspectives

Although plant nuclear proteomics is only at the initial phase, several studies have been reported using different species that include the analysis of the total nuclear or phospho-proteomes, and differential nuclear proteomes; however, few reports exist on the analysis of the maize nuclear proteome or its changes under various environmental conditions. In particular, most experiments have been done on the study of histones and their covalent modifications. The reason for this is probably due to the number of experiments done in epigenetics using this crop as a model, in particular in paramutation, imprinting, and transposon silencing. Thus, the low number of publications available provides most of the sources for this review. Therefore, the research presented here from other plant species can provide ideas for potential future experiments in maize. On the other hand, experiments using analysis by mass spectrometry, but also by immunological techniques will be very helpful to increase our knowledge of maize nuclear proteomics.

## Conflict of Interest Statement

The author declares that the research was conducted in the absence of any commercial or financial relationships that could be construed as a potential conflict of interest.
